# Improved Feedback Quantizer with Discrete Space Vector

**DOI:** 10.3390/s24010287

**Published:** 2024-01-03

**Authors:** Matías Veillon, Eduardo Espinosa, Pedro Melin, Galina Mirzaeva, Marco Rivera, Carlos R. Baier, Roberto O. Ramirez

**Affiliations:** 1 Department of Electrical Engineering, Faculty of Engineering, Universidad Católica de la Santísima Concepción, Talca 3467769, Chile; mveillon@magister.ucsc.cl; 2 Centro de Energía, Universidad Católica de la Santísima Concepción, Concepción 4090541, Chile; 3 Department of Electrical and Electronic Engineering, Universidad del Bío-Bío, Concepción 4051381, Chile; pemelin@ubiobio.cl; 4 School of Engineering, University of Newcastle, Callaghan, NSW 2308, Australia; galina.mirzaeva@newcastle.edu.au; 5 Power Electronics, Machines and Control Research Group, University of Nottingham, 15 Triumph Rd, Lenton, Nottingham NG7 2GT, UK; marco.rivera@nottingham.ac.uk; 6 Laboratorio de Conversión de Energía y Electrónica de Potencia (LCEEP), Vicerrectoría de Innovacion, Universidad de Talca, Curicó 3340000, Chile; 7 Department of Electrical Engineering, Faculty of Engineering, University of Talca, Curicó 3340000, Chile; cbaier@utalca.cl (C.R.B.); roramirez@utalca.cl (R.O.R.)

**Keywords:** Feedback Quantizer, Discrete Space Vector modulation, total harmonic distortion, weighted total harmonic distortion, voltage source converter, modulation scheme

## Abstract

The use of advanced modulation and control schemes for power converters, such as a Feedback Quantizer and Predictive Control, is widely studied in the literature. This work focuses on improving the closed-loop modulation scheme called Feedback Quantizer, which is applied to a three-phase voltage source inverter. This scheme has the natural behavior of mitigating harmonics at low frequencies, which are detrimental to electrical equipment such as transformers. This modulation scheme also provides good tracking for the voltage reference at the fundamental frequency. On the other hand, the disadvantage of this scheme is that it has a variable switching frequency, creating a harmonic spectrum in frequency dispersion, and it also needs a small sampling time to obtain good results. The proposed scheme to improve the modulation scheme is based on a Discrete Space Vector with virtual vectors to obtain a better approximation of the optimal vectors for use in the algorithm. The proposal improves the conventional scheme at a high sampling time (200 μs), obtaining a THD less than 2% in the load current, decreases the noise created by the conventional scheme, and provides a fixed switching frequency. Experimental tests demonstrate the correct operation of the proposed scheme.

## 1. Introduction

AC/DC or DC/AC power conversion is now essential because of the new changes in the energy matrix and the global context of generating clean energy. Thanks to technological advances, researchers have created various converter topologies and control schemes to meet the needs of each application [1,2,3,4,5]. These applications include AC motor drives [6], renewable energies [7], HVDC systems [8], electromobility [9], and microgrids [10].

The power converters work through a modulation scheme for the activation of semiconductors. For this, there are different techniques based on their applications or types of converters. Each technique is widely studied and focused on improving different characteristics, such as the total harmonic distortion (THD), the weighted harmonic distortion at low frequency (WTHD), the efficiency, power losses in semiconductors, and the computational load, among others [11,12].

This work focuses on the Feedback Quantizer (FBQ) modulation scheme, which is a closed-loop modulation scheme with the natural behavior of mitigating low-frequency harmonics, which are harmful to electrical equipment such as transformers [13,14]; in addition, it is good at tracking the voltage reference at the fundamental frequency [15], easy to implement, and also allows us to model the quantized noise with the introduction of filters [16,17,18,19]. This technique can be applied to microgrids or systems with transformers due to its low harmonic content at a low frequency, thus avoiding transformer saturation and increasing the equipment’s lifespan [20,21,22].

The FBQ modulation scheme is discrete and has characteristics similar to the Finite Control Set Model Predictive Control (FCS-MPC); it requires low system sampling times for high performance, generating a sparse harmonic spectrum and variable switching frequency [23,24,25,26].

So far, a slight improvement in the performance of the FBQ scheme has been achieved using the SVM modulation scheme, but it generates considerable noise at PWM voltages and still obtains a variable switching frequency [27].

The purpose of this work is to improve the FBQ modulation scheme at high sampling times by introducing the concept of a Discrete Space Vector (DSV) [28,29,30,31,32,33,34], which consists of the use of virtual vectors added to the conventional vector space of the converter to obtain a better choice of the optimal state to use, thus improving the accuracy of the technique at a high sampling time, and also setting the switching frequency to a fixed value equivalent to the system’s sampling time.

The structure of this document is as follows: In Section 2, we present the FBQ modulation scheme, showing its characteristics and simulating the system to be worked on. In Section 3, the improvement proposal using the DSV is presented, the system is simulated, and comparison indicators are shown to demonstrate the improvements in the technique. In Section 4, we proceed to the experimental results to validate the proposal. In Section 5, the advantages and disadvantage of the improved FBQ modulation scheme proposed are shown. Finally, in Section 6, the conclusions are presented.

## 2. Feedback Quantizer

**Figure 1:**
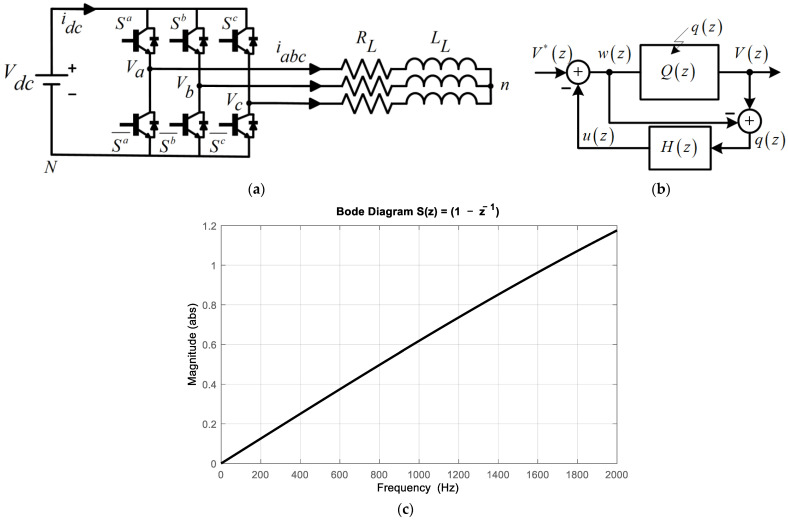
Conventional scheme: (**a**) two-level voltage source inverter (2L-VSI), (**b**) Feedback Quantizer scheme, and (**c**) Bode sensitivity diagram of the scheme.

The Feedback Quantizer (FBQ) is a closed-loop modulation scheme. Given its characteristics, it is possible to obtain an excellent harmonic spectrum in the PWM voltage of the inverter through this modulation, particularly the mitigation of low-frequency harmonics. The scheme is shown in Figure 1b, where $V^{*}(z)$ is the voltage reference, $V\left(z\right)$ is the quantized output voltage, $Q\left(z\right)$ represents the quantizer, q(z) is the quantized noise, H(z) is the feedback transfer function that will shape the system noise, and $u\left(z\right)$ and $w\left(z\right)$ are the quantized variables of the system.

The voltage reference is obtained by using the mathematical model of the voltage source inverter in Figure 1a, which is calculated with Clark coordinates and discretized by using the forward Euler method:

(1) $$V_{\alpha \beta }^{*}(kT)=\left(i_{\alpha \beta }(kT+T)-i_{\alpha \beta }(kT)\left[1-\frac{T_{s}R_{L}}{L_{L}}\right]\right)\frac{L_{L}}{T_{s}}.$$

The cost function ‘*g*’ to be used for the minimization of the switching states that will represent the quantizer $Q\left(z\right)$ as:

(2) $$g=\sqrt{{\left(V_{\alpha }^{*}-V_{i\alpha }\right)}^{2}+{\left(V_{\beta }^{*}-V_{i\beta }\right)}^{2}},$$

where $\left(V_{\alpha }^{*},V_{\beta }^{*}\right)$ and $\left(V_{i\alpha },V_{i\beta }\right)$ are the voltages in Clark coordinates of the reference and switching state at instant *i,* respectively, where *i =* (0, 1, 2…7). A two-level voltage source inverter has six valid switching states and two nulls. Table 1 shows the possible states of the inverter.

Then, the equation of the output variable of the scheme can be written as follows:

(3) $$V(z)=V^{*}(z)+q(z)-q(z)=V^{*}(z)$$

From (3), if *H*(*z*) = 1, we have perfect quantized voltage tracking, but this is not possible due to causality; therefore, the optimal *H*(*z*) to use will be $z^{-1}$, which has several advantages, such as ease of system analysis, the natural behavior of the system for low-frequency harmonic mitigation, and easy implementation of notch filters [16,17]. Therefore, the equation of the system is as shown in Equation (4) and replacing *H*(*z*) = $z^{-1}$ in (4), we obtain Equation (5).

(4) $$V(z)=V^{*}(z)+(1-H(z))q(z)$$

(5) $$V(z)=V^{*}(z)+\left(\frac{z-1}{z}\right)q(z)$$

From (5), we can obtain the transfer function of the system sensitivity *S*(*z*) (6), and using the optimal *H*(*z*), we have (5):

(6) S(z)=1−H(z)

(7) $$S(z)=1-z^{-1}.$$

For the analysis of the sensitivity system at the frequency plane, $z=e^{j\omega_{n}}$, the magnitude of the system can be obtained:

(8) $$|1-H(e^{j\omega_{n}})|=\sqrt{2-2\cos(\omega_{n})}.$$

From the magnitude obtained in (8) and Figure 1c, we can observe the natural behavior of the FBQ system, where it tends to mitigate the low-frequency harmonics, and, in addition, good tracking of the voltage reference at the fundamental frequency (50 Hz) is obtained. Then, it can be observed from (5), replacing *H*(*z*) with $z^{-1}$, that by having a zero at *z* = 1 the system will not have DC components in the voltages produced by the converter.

Next, we will show some brief simulations of the FBQ scheme, considering the parameters in Table 2.

**Figure 2:**
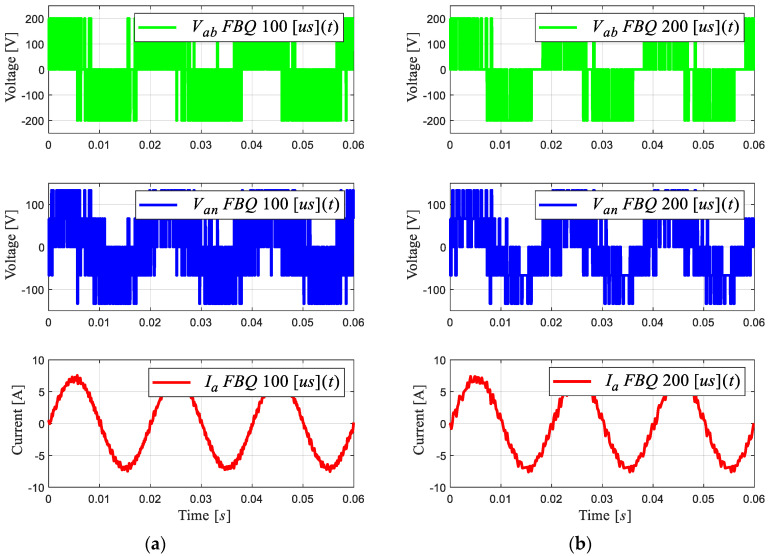
PWM voltages and current phase “a” of 2L-VSI: (**a**) FBQ 100 [μs], (**b**) FBQ 200 [μs].

**Figure 3:**
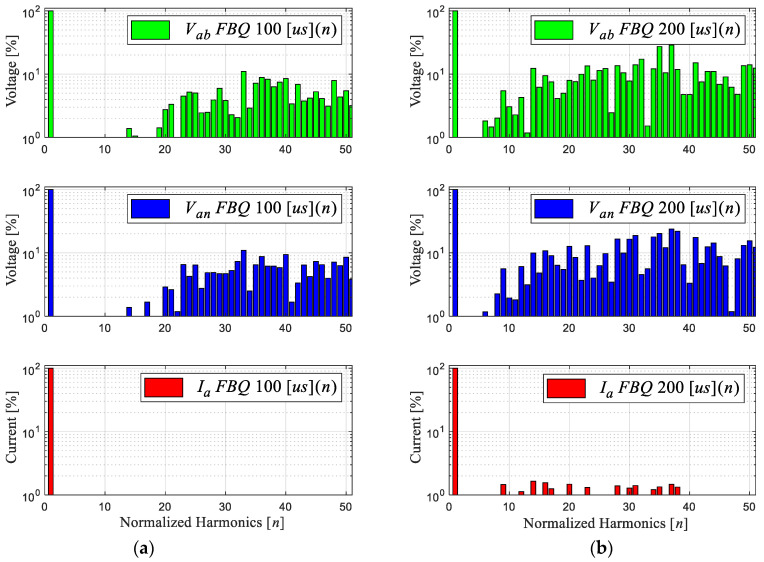
Harmonic spectrum of signals: (**a**) FBQ 100 [μs] and (**b**) FBQ 200 [μs].

Figure 2 shows the FBQ simulation for different sampling times, namely, 100 [μs] and 200 [μs], where the PWM voltages and the current phase “a” are shown for 2L-VSI with their respective harmonic spectra (shown in Figure 3). The harmonic spectrum has a low-order harmonic mitigation; as observed in the Bode diagram in Figure 1c, the low-frequency harmonics in the PWM voltages (line to line and line to neutral) are mitigated. In Figure 3a, these begin to show in approximately the 12th harmonic, with an amplitude of 1% to its fundamental amplitude; after the 20th harmonic, its magnitude exceeds 10%. Then, by increasing the sampling time to 200 μs (Figure 3b), a more significant distortion of these signals can be observed, and the magnitude of the harmonics increases, with more harmonic components showing at low frequencies. This is due to the high sampling time, generating a deficient reference voltage because there are insufficient data, and the choice of the optimal states has a greater range of error.

The objective of this work is to improve the performance of the FBQ modulation scheme at high sampling times, in this case at 200 μs, with the use of DSV.

## 3. Proposal for a Feedback Quantizer with a Discrete Space Vector

**Figure 4:**
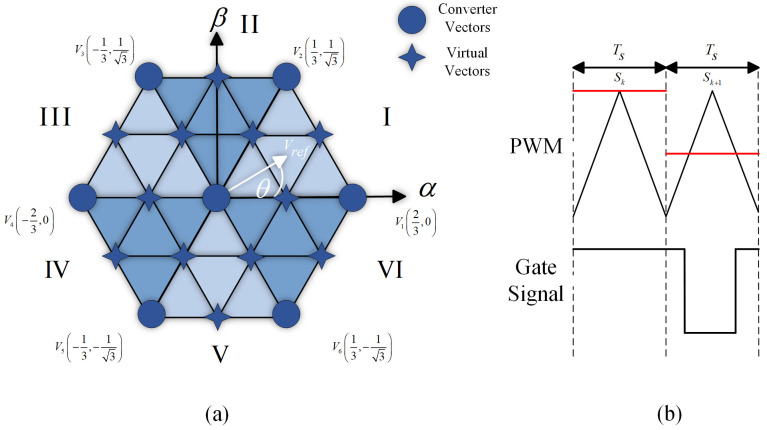
Discrete Space Vector: (**a**) vector space with 12 virtual vectors; (**b**) approximation of virtual to real vectors by using PWM.

A Discrete Space Vector is a technique to approximate the choice of finite states in discrete systems more accurately, using virtual vectors in addition to the system’s real vectors. When implementing it in the FBQ modulation scheme, the totality of the vectors (real and virtual) must be considered in the vector of the possible states. Then, the cost function will iterate the number of vectors to obtain the closest one; for example, in Figure 4a, we have defined 12 virtual vectors. Therefore, the system iteration will be 19 times, considering the sum of the real vectors; then, the approximation of the virtual vector is made with PWM, as shown in Figure 4b.

**Figure 5:**
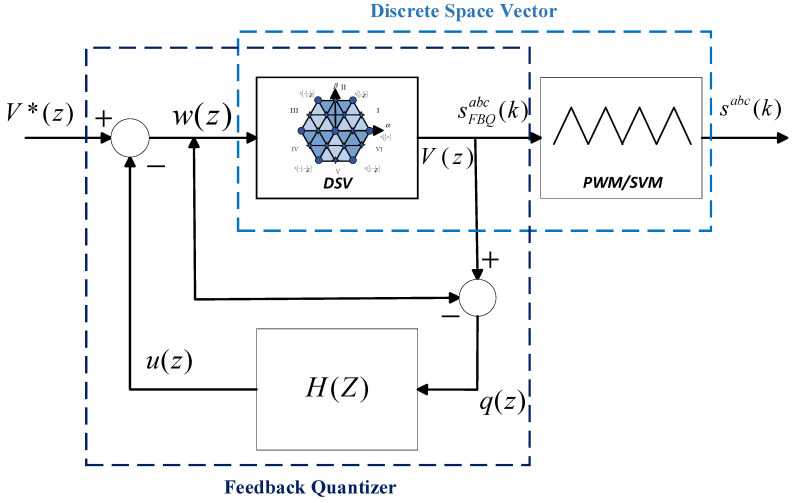
Feedback Quantizer with Discrete Space Vector.

**Figure 6:**
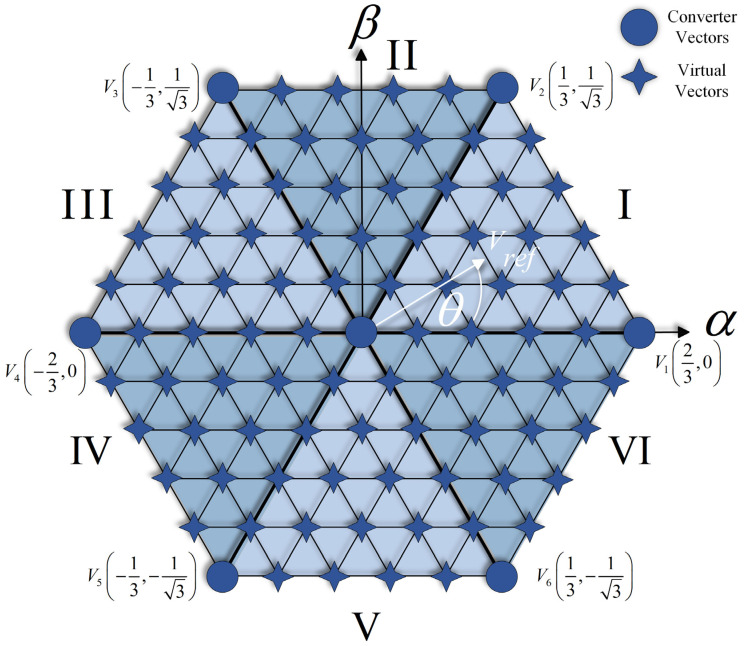
Space Vector of the converter with 91 vectors.

Then, in Figure 5, the complete diagram is shown using DSV in the FBQ modulation scheme. In the proposed scheme, the more virtual vectors are defined, the higher the accuracy of the choice of the optimal vector to be chosen in the algorithm; therefore, a better result is obtained in terms of wave quality (WTHD and THD) and reference tracking. By increasing the number of virtual vectors, the computational load increases considerably; therefore, due to hardware limitations, we will work with 84 virtual vectors (91 vectors in total, Figure 6).

**Figure 7:**
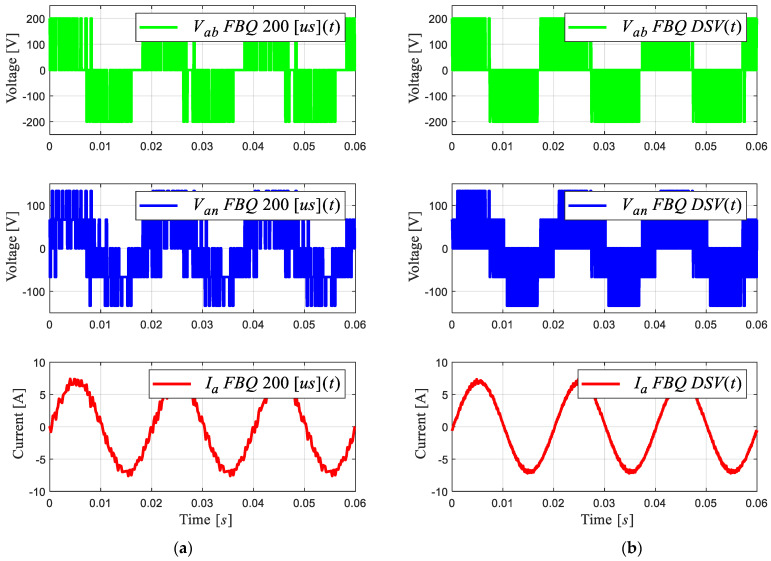
PWM voltages and current phase “*a*” of 2L-VSI: (**a**) FBQ 200 [μs]; (**b**) FBQ DSV 200 [μs].

**Figure 8:**
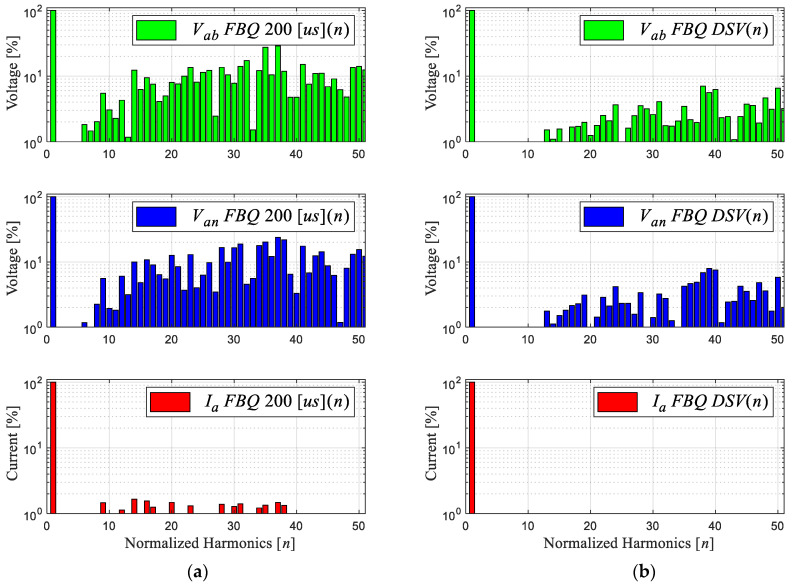
Harmonic spectrum of signals: (**a**) FBQ 200 [μs] and (**b**) FBQ DSV 200 [μs].

Next, Figure 7 shows the simulation of the proposed method, noting the improvement in the scheme at a sampling time of 200 μs. Figure 7a shows the conventional scheme (same Figure 2b), and Figure 7b shows the improved scheme with the DSV. A clear improvement in the wave quality of the obtained signals and a considerable reduction in the magnitude of the harmonic components can be observed in Figure 8b. In addition, a fixed switching frequency equivalent to the sampling time used is obtained; this is due to the approximation of the virtual to real vectors, by means of the triangular signal with a frequency equivalent to the sampling time, i.e., 5 [kHz].

Table 3 shows the summary of the indicators obtained from the simulations, first of all noting the sampling change differences between THD and WTHD in the conventional FBQ system. Also, the tracking of the voltage reference is obtained, where good results are confirmed (higher than 99%, i.e., the scheme’s ability to follow the voltage reference at fundamental frequency has an error of less than 1%); this is obtained by comparing the fundamental components between the FBQ schemes, obtained using the harmonic spectrum and the fundamental component of the classical SPWM modulation. The indicators corresponding to the proposed scheme are shown in the third column of Table 3, with better results obtained at a higher sampling time than the two previous simulations. In addition, it is noted that the fixed switching frequency is equivalent to the system’s sampling time. The switching frequency of the system was calculated by taking the trigger pulses of each phase. (*S_a_*, *S_b_* y *S_c_*) for ten signal periods and averaged together (9).

(9) $$f_{s}=\frac{\left(\frac{S_{a}}{n{}^{\circ}\;Periods}*f_{fundamental}\left(50\;hz\right)+\frac{S_{b}}{n{}^{\circ}\;Periods}*f_{fundamental}\left(50\;hz\right)+\frac{S_{c}}{n{}^{\circ}\;Periods}*f_{fundamental}\left(50\;hz\right)\right)}{3},$$

## 4. Experimental Results

**Figure 9:**
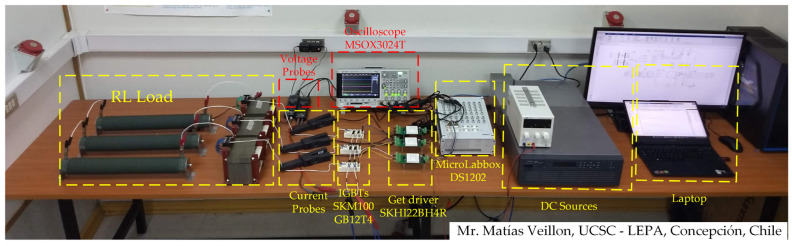
Experimental setup.

A three-phase two-level inverter was assembled to validate the proposed modulation scheme. The experimental prototype is shown in Figure 9, where a MicroLabBox dSPACE 1202 was used for the digital processing. The system parameters were the same as those used in the simulation results, as shown in Table 2.

**Figure 10:**
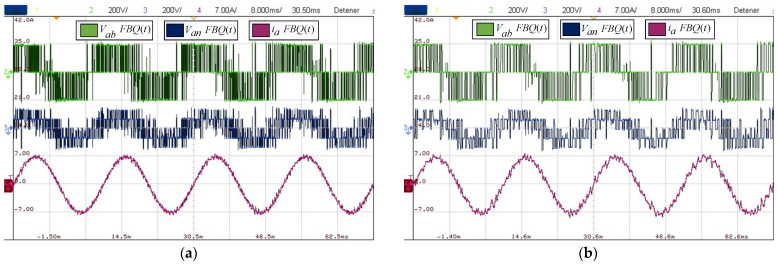
Experimental results: (**a**) FBQ 100 [μs]; (**b**) FBQ 200 [μs].

Next, the same procedure seen in the simulations was performed, obtaining steady-state signals and visualizing the conventional FBQ scheme when the sampling time changed. Figure 10a,b show that the signals immediately have more distortion at a higher sampling time. This is mentioned above because the system’s reference depends on the sampling time, and by increasing this, the data decreases to have a more accurate representation of the reference, and the switching frequency decreases with this change in sampling time.

**Figure 11:**
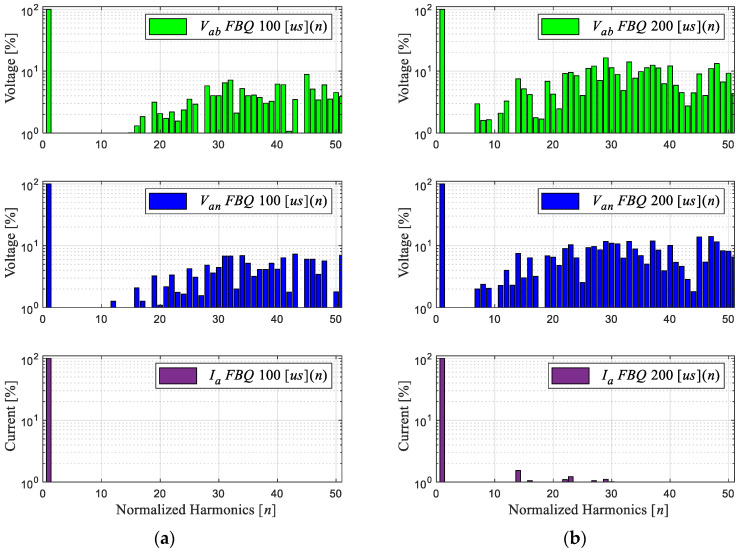
Experimental results (harmonic spectrum): (**a**) FBQ 100 [μs]; (**b**) FBQ 200 [μs].

For the distortion indicators for FBQ at 100 [μs], we have a THD of the PWM voltages of 35% and current THD of 2.48%, while for the weighted distortion at low frequency, we have a WTHD of 0.75% for the voltages and a WTHD of 0.08% for the current. Then, by analyzing the same indicators at a higher sampling time, in this case, at 200 [μs], we can appreciate the increase in the indicators, obtaining a voltage THD of 50% and a current THD of 5%, a WTHD of 1.74%, and, for the current, a WTHD of 0.28%. This is also reflected in the harmonic spectrum in Figure 11b, where we can see the increase in the magnitudes of the harmonic components in the PWM voltages and the presence of harmonics greater than 1% in the phase current.

**Figure 12:**
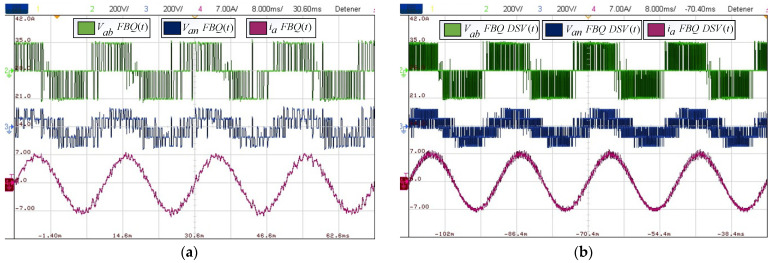
Experimental results: (**a**) FBQ 200 [μs]; (**b**) FBQ-DSV 200 [μs].

**Figure 13:**
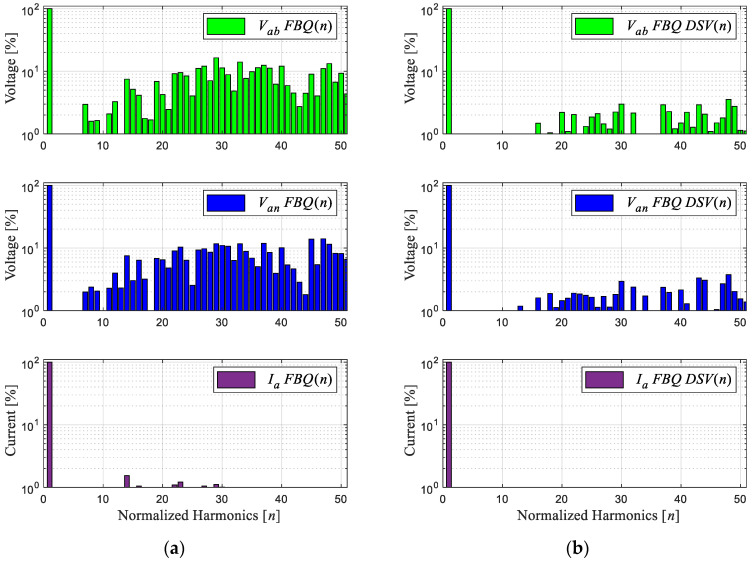
Experimental results of the harmonic spectrum: (**a**) FBQ 200 [μs]; (**b**) FBQ-DSV 200 [μs].

Next, we obtained the experimental results of the proposed scheme by comparing the results of the conventional and proposed schemes at a sampling time of 200 [μs]. Figure 12b shows that the waveforms obtained are much more defined compared to the conventional FBQ modulation at the identical sampling time (Figure 12a). This can also be observed in the harmonic aspect of the voltages of both results. In Figure 13b, the proposed scheme obtains considerably lower magnitudes of the harmonics (no more than 4%), while the conventional method has magnitudes of harmonics higher than 10%, and more harmonics components are present at lower frequencies in the signal’s spectrums (Figure 13a).

Regarding the distortion indicators obtained, for the PWM voltages, there is a THD of 11.19%, while for the phase current, there is a THD of 1.28%; concerning the weighted distortion indicators at low frequency, there is a voltage and current WTHD of 0.41% and 0.11%, respectively.

**Figure 14:**
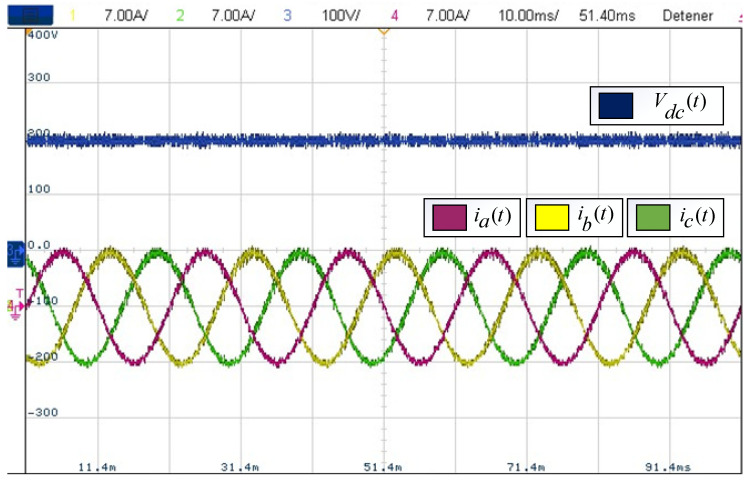
Balanced three-phase currents and DC voltage.

Finally, a summary of the indicators measured up to the 51st harmonic obtained from the experimental results is shown in Table 4. This is in exchange for an increase in the switching frequency equal to the scheme’s sampling frequency. Finally, Figure 14 shows the steady-state balanced three-phase currents of the system and its DC supply voltage.

## 5. Advantages and Disadvantages of the Proposed Scheme

The proposed scheme allows us to improve the conventional FBQ scheme at high sampling times, where discrete schemes have a low performance (for this type of technique, high sampling times above 100 μs and low sampling times below 50 μs are considered). Also, a fixed switching frequency is obtained in comparison to the conventional scheme that delivers a variable switching frequency. Finally, it should be noted that the DSV technique takes into account the discrete nature of the converter, without affecting the natural behavior of the FBQ scheme. Regarding the disadvantages of the technique, it is the computational cost; the major computational cost of the technique focuses on the iterative cycle to minimize the cost function. Thus, if the conventional FBQ scheme iterates eight times, the proposed scheme iterates 91 times, increasing the computational load by slightly more than 11 times. Finally, the switching frequency increases in synchronization with the sampling frequency of the system.

## 6. Conclusions

An improvement to the Feedback Quantizer modulation scheme with a Discrete Space Vector applied to a three-phase two-level inverter was presented in this study. It was possible to couple the DSV technique to the FBQ modulation scheme to improve the wave quality of the signals at a high sampling time, in this case, 200 [μs], and to obtain a fixed switching frequency equivalent to the system’s sampling time. The conventional FBQ scheme at 100 [μs] obtained a 35.6% THD voltage and a 2.4% THD current; this was calculated up to the 51st harmonic. The proposed scheme at 200 [μs], with 91 vectors improves the wave quality, obtaining an 11.1% THD voltage and a 1.2% THD load current.

This fulfills the objectives of improving the conventional FBQ scheme at high system sampling times and obtaining a fixed switching frequency, solving one of the problems highlighted in discrete character schemes. Both the theoretical and experimental results demonstrate the correct operation of the proposed scheme.

## Figures and Tables

**Table 1:** Valid states of the voltage source inverter.

Vector	*a*	*b*	*c*	α	β
$V_{0}$	0	0	0	0	0
$V_{1}$	1	0	0	2/3	0
$V_{2}$	1	1	0	1/3	$1/\sqrt{3}$
$V_{3}$	0	1	0	−1/3	$1/\sqrt{3}$
$V_{4}$	0	1	1	−2/3	0
$V_{5}$	0	0	1	−1/3	$-1/\sqrt{3}$
$V_{6}$	1	0	1	1/3	$-1/\sqrt{3}$
$V_{7}$	1	1	1	0	0

**Table 2:** Simulation parameters 2L-VSI.

Symbol	Name	Value
$R_{L}$	Load Resistance	10 [Ω]
$L_{L}$	Load Inductance	15 [mH]
$V_{dc}$	DC Voltage Link	200 [V]
$T_{s}$	Sampling Time	100 [μs]/200 [μs]

**Table 3:** Harmonic distortion indicators summary of comparison indicators.

	FBQ 100 [μs]	FBQ 200 [μs]	FBQ 200 [μs] 91 Vectors
THD Ia [%]	2.81	5.2	1.23
THD Va [%]	39.06	45.16	16.17
WTHD Ia [%]	0.083	0.30	0.0643
WTHD Van [%]	1.06	2.27	0.5021
Switching Frequency [Hz]	2500	1200	5000
Voltage Reference Tracking [%]	99.74	99.76	99.81

**Table 4:** Harmonic Distortion Indicators.

	FBQ 100 [μs]	FBQ 200 [μs]	FBQ 200 [μs] 91 Vectors
THD Ia [%]	2.4822	5.0631	1.2802
THD Van [%]	35.6874	50.8163	11.1990
WTHD Ia [%]	0.0881	0.2874	0.1106
WTHD Van [%]	0.7577	1.7429	0.4171

## Data Availability

Data are contained within the article.
